# Uncovering the Hidden Diversity and Antimicrobial Resistance of Uropathogens in a Tertiary‐Care Hospital in Bangladesh

**DOI:** 10.1155/ijm/8327078

**Published:** 2026-07-09

**Authors:** Md. Arman Hosen, Tanzim Rahman, Rahatuzzaman, Rubaiya Binte Kabir, Chowdhury Rafiqul Ahsan, Mustafizur Rahman, Mahmuda Yasmin, Mohammad Jubair

**Affiliations:** ^1^ Genome Centre, Infectious Diseases Division, International Centre for Diarrhoeal Disease Research, Bangladesh (icddr, b), Dhaka, Bangladesh, icddrb.org; ^2^ Laboratory of Environmental Health, Health Systems and Population Studies Division, International Centre for Diarrhoeal Disease Research, Bangladesh (icddr, b), Dhaka, Bangladesh, icddrb.org; ^3^ Department of Urology, Dhaka Medical College and Hospital, Dhaka, Bangladesh, dmc.edu.bd; ^4^ Department of Microbiology, University of Dhaka, Dhaka, Bangladesh, du.ac.bd

**Keywords:** 16S rRNA sequencing, AMR, Bangladesh, fosfomycin, metagenomics, nitrofurantoin, urinary microbiome, urinary tract infection, uropathogens

## Abstract

Urinary tract infections (UTIs) are among the most common bacterial infections worldwide; however, their diagnosis in low‐ and middle‐income countries often relies on conventional culture and biochemical methods with limited sensitivity. This study evaluated the limitations of routine diagnostic approaches and explored the microbial diversity and antimicrobial resistance (AMR) profiles of uropathogens in a tertiary‐care hospital in Bangladesh using integrated culture‐based and molecular methods. Among 30 patient urine samples collected in 2025, 10 were selected for detailed analysis due to funding and resource limitations; therefore, the findings should be interpreted as exploratory and may be subject to selection bias. Of these 10 samples, routine hospital diagnostics identified only eight isolates, whereas extended biochemical analysis detected 29 isolates, indicating substantial underestimation of microbial diversity in standard practice. Antibiotic susceptibility testing revealed a high prevalence of multidrug resistance, with 83% and 80% of isolates resistant to ampicillin and clindamycin, respectively. In contrast, nitrofurantoin and fosfomycin retained effectiveness against most isolates, supporting their continued clinical utility. 16S rRNA gene sequencing further revealed complex and heterogeneous microbial communities, with several samples dominated by *Escherichia-Shigella*, whereas others exhibited polymicrobial profiles including commensal and opportunistic genera. Despite taxonomic variability, microbial diversity did not differ significantly between inpatient and outpatient groups. Functional pathway prediction demonstrated a largely conserved metabolic profile across samples, including pathways associated with virulence, iron acquisition, and AMR. Overall, this study demonstrates that conventional diagnostic methods substantially underestimate uropathogen diversity and may contribute to misdiagnosis and inappropriate antibiotic use. Integrating molecular approaches into routine clinical workflows could improve pathogen detection, enhance AMR surveillance, and support more effective management of UTIs in Bangladesh and similar resource‐limited settings.

## 1. Introduction

Urinary tract infections (UTIs) are among the most prevalent bacterial infections worldwide, affecting an estimated 404.6 million people annually and accounting for approximately 236,790 deaths globally in 2019 [1, 2]. The incidence of UTIs has risen markedly over recent decades, with a 60.4% increase in reported cases since 1990, underscoring the growing public health significance of these infections worldwide [2]. In low‐ and middle‐income countries (LMICs), including Bangladesh, the burden is further compounded by limited diagnostic infrastructure, widespread over‐the‐counter antibiotic use, and the emergence of multidrug‐resistant (MDR) uropathogens [3, 4]. UTI is also becoming a major public health problem in Bangladesh, with many people experiencing repeated infections and unsuccessful treatments, and about 65% of the bacterial pathogens causing these infections are resistant to multiple antibiotics [5]. Uropathogenic *E. coli* is the leading cause of both uncomplicated and complicated UTIs worldwide, whereas other pathogens like *Enterococcus*, *Staphylococcus*, *Pseudomonas*, and *Candida* species contribute more prominently to complicated and healthcare‐associated infections [6, 7]. UTIs cover various health issues, from urethritis and cystitis (lower tract) to serious pyelonephritis (upper tract), usually caused by infections that move upward and a higher load of bacteria affecting the kidneys [8–10]. For a long time, physicians have utilized Kass′s standard of ≥ 10^5^ colony‐forming units per milliliter (CFU/mL) from clean‐catch urine samples to read urine cultures [11, 12]. However, new research and guidelines for children show that lower levels are acceptable [13]. Despite advances in clinical microbiology, the diagnosis of UTIs still largely depends on culture‐based methods, which have limited sensitivity for detecting fastidious, slow‐growing, or low‐abundance organisms [14]. These limitations may lead to underestimation of microbial diversity and failure to detect polymicrobial infections, ultimately contributing to misdiagnosis and inappropriate treatment [15].

Uropathogens are increasingly able to resist antibiotics in different ways, such as by making *β*‐lactamase, using efflux pumps, changing their targets, and sharing resistance genes [16, 17]. For instance, extended‐spectrum *β*‐lactamase (ESBL)–producing *Escherichia coli* and *Klebsiella pneumoniae* demonstrate resistance to third‐generation cephalosporins, whereas carbapenem‐resistant *Pseudomonas aeruginosa* and *Acinetobacter* spp. further limit treatment options [18–20]. In the same way, resistance to trimethoprim‐sulfamethoxazole mediated by *dfr* genes and fluoroquinolone resistance due to *gyrA* mutations highlight the evolving antimicrobial resistance (AMR) landscape among UTI‐causing pathogens [21–23]. The rapid emergence and dissemination of AMR among uropathogens have further complicated treatment strategies, particularly in LMICs where antibiotic misuse and limited diagnostic capacity are common [24]. This underscores the urgent need for more sensitive and comprehensive diagnostic approaches to guide targeted therapy and reduce inappropriate antibiotic use.

Recent advances in molecular techniques, particularly 16S rRNA gene sequencing, have enabled culture‐independent profiling of microbial communities, providing a more comprehensive understanding of microbial diversity, including unculturable organisms [25, 26]. These approaches offer deeper insights into the urinary microbiome and its role in disease pathogenesis.

This exploratory single‐center study aimed to assess the limitations of routine diagnostic approaches and characterize the bacterial diversity and AMR profiles of uropathogens among selected urine samples collected from a tertiary‐care hospital in Dhaka, Bangladesh. This study is aimed at addressing an essential diagnostic deficiency in UTI management: conventional clinical biochemical techniques frequently exhibit insufficient sensitivity and specificity, potentially overlooking the actual diversity of uropathogens, hence leading to misdiagnosis and improper antibiotic application. Routine urine culture in most Bangladeshi hospitals relies on chromogenic agar and only a limited set of biochemical tests, which primarily target common pathogens such as *E. coli* and *Klebsiella*. As a result, slow‐growing, fastidious, or less prevalent bacteria—many of which can cause complicated or persistent infections—remain undetected [15]. In this study, routine hospital diagnostics refers to the standard clinical urine culture and preliminary identification performed at Dhaka Medical College Hospital. Laboratory‐based culture refers to repeat culture on MacConkey and blood agar, whereas extended biochemical analysis refers to further identification of recovered colonies using standard biochemical tests.

To highlight the limitations of conventional biochemical identification methods, this study employed 16S rRNA sequencing for comprehensive microbial profiling. Although phenotypic antimicrobial susceptibility testing (AST) was used to assess AMR profiles of cultured isolates, 16S rRNA gene sequencing was included as a complementary culture‐independent method to characterize bacterial community composition and identify microbial diversity that may be overlooked by routine diagnostic culture. Because 16S rRNA sequencing targets a conserved taxonomic marker, it was not intended to directly detect AMR genes or distinguish resistant from susceptible strains. Additionally, functional pathway prediction was performed to explore virulence and resistance‐associated mechanisms, enabling a shift from purely taxonomic identification to functional interpretation of microbial communities.

Overall, this study highlights the importance of integrating molecular and genomic tools into routine diagnostics to improve detection accuracy, enhance AMR surveillance, and inform more effective clinical management strategies for UTIs in Bangladesh and similar settings.

## 2. Materials and Methods

### 2.1. Sample Collection and Bacterial Isolation

A total of 30 urine samples were collected from patients admitted to Dhaka Medical College Hospital, Dhaka, Bangladesh in 2025. Patients were eligible for inclusion if they were admitted as inpatients or attended the outpatient department of Dhaka Medical College Hospital with suspected UTI and provided a urine sample for routine diagnostic evaluation (Table 1). Both culture‐positive and culture‐negative patient samples were considered for further analysis to compare routine diagnostic findings with extended laboratory‐based and molecular approaches. Healthy volunteer samples were included as controls if donors had no reported symptoms of UTI at the time of sample collection. Samples were excluded if the available urine volume was insufficient for laboratory processing, if the sample was visibly contaminated or improperly stored, or if essential metadata such as age, sex, clinical status, or collection source were unavailable. Urine samples were collected using the midstream clean‐catch method whenever possible, following standard clinical collection procedures. Because of funding constraints, 10 patient samples and 4 healthy control samples were selected for detailed analysis. The 10 patient samples were obtained from individuals aged 19–65 years, including 7 males and 3 females. This subset was not designed to be sex‐stratified and therefore may not reflect the expected higher burden of UTIs among females. Four samples were from inpatients and six were from outpatients. Data on prior antibiotic exposure were not available, which may limit interpretation of the observed microbial diversity and AMR patterns.

**Table 1:** Metadata of collected urine samples and those included in the study.

Sample ID	Group	Age (years)	Sex	Clinical status	Collection source	Detailed analysis
1C	Control	25	Male	Healthy	University of Dhaka	This study
2C	Control	26	Male	Healthy	University of Dhaka	This study
3C	Control	25	Male	Healthy	University of Dhaka	This study
4C	Control	25	Male	Healthy	University of Dhaka	This study
2A	Patient	33	Female	Culture‐negative^a^	Outpatient	
3A	Patient	40	Male	Culture‐positive^b^	Outpatient	
4A	Patient	40	Male	Culture‐positive	Inpatient	
5A	Patient	32	Female	Culture‐positive	Inpatient	
6A	Patient	70	Female	Culture‐positive	Inpatient	
7A	Patient	70	Male	Culture‐negative	Inpatient	
9A	Patient	54	Female	Culture‐positive	Inpatient	
11A	Patient	34	Female	Culture‐positive	Outpatient	
14A	Patient	12	Female	Culture‐negative	Inpatient	
15A	Patient	42	Male	Culture‐positive	Inpatient	
16A	Patient	55	Male	Culture‐positive	Outpatient	This study
17A	Patient	54	Female	Culture‐positive	Outpatient	This study
18A	Patient	48	Male	Culture‐positive	Outpatient	This study
19A	Patient	65	Male	Culture‐positive	Inpatient	This study
20A	Patient	22	Male	Culture‐positive	Inpatient	This study
21A	Patient	33	Female	Culture‐negative	Inpatient	This study
22A	Patient	19	Male	Culture‐negative	Outpatient	This study
23A	Patient	45	Male	Culture‐positive	Outpatient	This study
24A	Patient	26	Female	Culture‐negative	Inpatient	This study
25A	Patient	37	Male	Culture‐negative	Outpatient	This study
26A	Patient	41	Male	Culture‐positive	Outpatient	
27A	Patient	32	Male	Culture‐positive	Outpatient	
28A	Patient	56	Female	Culture‐positive	Outpatient	
29A	Patient	60	Female	Culture‐positive	Inpatient	
30A	Patient	34	Male	Culture‐negative	Inpatient	
31A	Patient	49	Female	Culture‐positive	Outpatient	
32A	Patient	34	Female	Culture‐positive	Inpatient	
36A	Patient	23	Male	Culture‐negative	Inpatient	
37A	Patient	35	Male	Culture‐positive	Inpatient	
38A	Patient	37	Female	Culture‐positive	Inpatient	

^a^Culture‐negative (for samples with no detectable growth).

^b^Culture‐positive (for samples with bacterial growth).

Approximately 0.5 mL of each sample was inoculated into nutrient broth and incubated for 3 h to facilitate bacterial recovery. Subsequently, a loopful of culture was streaked onto MacConkey agar and blood agar plates and incubated at 37°C for 18–24 h. Distinct colonies were selected based on morphological characteristics and subjected to a panel of standard biochemical tests, including Kligler Iron Agar (KIA), citrate utilization, catalase, oxidase, indole, and motility‐indole‐urease (MIU) tests for presumptive identification.

### 2.2. AST

AST was performed using the disk diffusion method in accordance with Clinical and Laboratory Standards Institute (CLSI) guidelines. The antibiotic disk contents used in this study represented standardized disk potencies for each antimicrobial agent rather than different experimental dosages. The tested antibiotics included ampicillin (10 *μ*g), amoxicillin (10 *μ*g), amoxicillin‐clavulanate (30 *μ*g), clindamycin (2 *μ*g), ceftazidime (30 *μ*g), imipenem (10 *μ*g), gentamicin (30 *μ*g), tetracycline (30 *μ*g), norfloxacin (10 *μ*g), nitrofurantoin (300 *μ*g), trimethoprim (5 *μ*g), and fosfomycin (200 *μ*g). Zones of inhibition were measured in millimeters and interpreted as susceptible, intermediate, or resistant according to CLSI breakpoints. CLSI interpretive criteria were applied using species‐level identification where available. For isolates identified only at the genus level, applicable genus‐level or organism‐group CLSI criteria were used where available.

### 2.3. DNA Extraction and 16S rRNA Sequencing

Genomic DNA was extracted from urine samples from patients (*n* = 10) using the DNeasy Blood & Tissue Kit (QIAGEN, Hilden, Germany) following the manufacturer′s protocol. Healthy volunteer samples were included only as culture‐based controls and were not subjected to 16S rRNA sequencing. Blank extraction controls and no‐template PCR controls were included during DNA extraction and 16S rRNA gene amplification to monitor potential contamination. No amplification was observed in the negative controls. The V4 hypervariable region of the bacterial 16S rRNA gene was amplified following the Earth Microbiome Project (EMP) protocol using the universal primer pair 515F/806R with Illumina adapter overhang sequences. PCR amplification was performed under EMP‐recommended cycling conditions to ensure consistency and minimize bias. Amplicons were purified using AMPure XP magnetic beads to remove primers and small fragments. The libraries were purified, quantified using a Qubit fluorometer, normalized, and pooled in equimolar concentrations. The final library pool was sequenced on the Illumina MiSeq platform using paired‐end (2 × 250 bp) chemistry according to the manufacturer′s instructions.

### 2.4. Bioinformatic and Statistical Analysis

Raw sequencing reads were processed using the DADA2 pipeline (v1.10.0) in R (v3.5.1), including quality filtering, denoising, chimera removal, paired‐end read merging, and generation of amplicon sequence variants (ASVs) [25]. Filtering parameters included truncLen = c(240,160), maxN = 0, maxEE = c [2], and truncQ = 2. Samples with insufficient sequencing depth or poor‐quality reads after filtering were excluded from downstream analysis. The final ASV table was normalized to even sequencing depth before diversity and taxonomic analyses.

Taxonomic classification was performed using the SILVA v138.2 database [27]. Data visualization was conducted using ggplot2 [28].

Microbiome analysis was conducted using the phyloseq package [26]. Alpha diversity (Chao1, Shannon, Simpson) was calculated using estimate_richness(), and differences were assessed using the Wilcoxon rank‐sum test. Beta diversity was evaluated using Bray–Curtis dissimilarity and visualized by principal coordinates analysis (PCoA). Statistical differences were assessed using permutational multivariate analysis of variance (PERMANOVA) (adonis2 function, vegan package).

Functional prediction was performed using PICRUSt2, with pathway annotation based on the MetaCyc database [29, 30]. Differential pathway analysis was conducted using the ALDEx2 method via ggpicrust2. Statistical significance was determined using the Wilcoxon test with Benjamini–Hochberg correction (adjusted *p* < 0.05). Heatmaps were generated using the pheatmap package following Z‐score normalization.

## 3. Result

### 3.1. Comparative Culture Between Hospital and Laboratory

Comparative culture analysis revealed notable differences between routine hospital diagnostics and laboratory‐based culture results (Table 2). Several samples categorized as culture‐negative in hospital diagnostics demonstrated bacterial growth under laboratory conditions, particularly on blood agar, indicating potential under‐detection in routine clinical workflows.

**Table 2:** Culture results of urine samples on selective media in Dhaka University laboratory.

Sample ID	Collection source	Clinical status	Laboratory culture	
			MacConkey agar	Blood agar
1C	University of Dhaka	Healthy	−	+
2C	University of Dhaka	Healthy	−	−
3C	University of Dhaka	Healthy	−	−
4C	University of Dhaka	Healthy	−	−
16A	Outpatient	Culture‐positive	−	+
17A	Outpatient	Culture‐positive	−	+
18A	Outpatient	Culture‐positive	−	+
19A	Inpatient	Culture‐positive	−	+
20A	Inpatient	Culture‐positive	+	+
21A	Outpatient	Culture‐negative	−	+
22A	Outpatient	Culture‐negative	+	+
23A	Outpatient	Culture‐positive	+	+
24A	Inpatient	Culture‐negative	−	+
25A	Outpatient	Culture‐negative	+	+

Overall, bacterial growth was detected in 78.6% (11/14) of samples on blood agar compared with only 28.6% (4/14) on MacConkey agar. All culture‐positive patient samples showed growth on blood agar, whereas only a subset exhibited growth on MacConkey agar, suggesting that not all isolates were enteric gram‐negative bacteria. Notably, samples initially classified as culture‐negative (21A, 22A, 24A, and 25A) showed positive growth upon laboratory culture. Statistical comparison using McNemar′s test demonstrated that blood agar detected significantly more bacterial growth than MacConkey agar (*p* = 0.008).

### 3.2. Isolation and Characterization of Uropathogens

A total of 10 urine samples collected from patients were analyzed, of which all samples showed visible bacterial growth, yielding a total of 29 isolates. Among the four samples collected from healthy volunteers, only one exhibited bacterial growth on blood agar.

Biochemical characterization revealed that the majority of isolates produced acid and gas in the KIA test, whereas a smaller proportion produced hydrogen sulfide (H_2_S). Indole production was observed in a subset of isolates, facilitating differentiation among enteric bacteria. Only a limited number of isolates demonstrated oxidase‐positive activity.

Based on biochemical identification, *E. coli* was the most frequently isolated organism (*n* = 8), followed by *Enterobacter* spp. (*n* = 6) and *Proteus* spp. (*n* = 6). Other identified organisms included *Pseudomonas* spp. (*n* = 3), *K. pneumoniae* (*n* = 2), *Morganella* spp. (*n* = 2), *Staphylococcus aureus* (*n* = 2), and *Acinetobacter* spp. (*n* = 1).

### 3.3. AMR Pattern of the Identified Isolates

**Figure 1:**
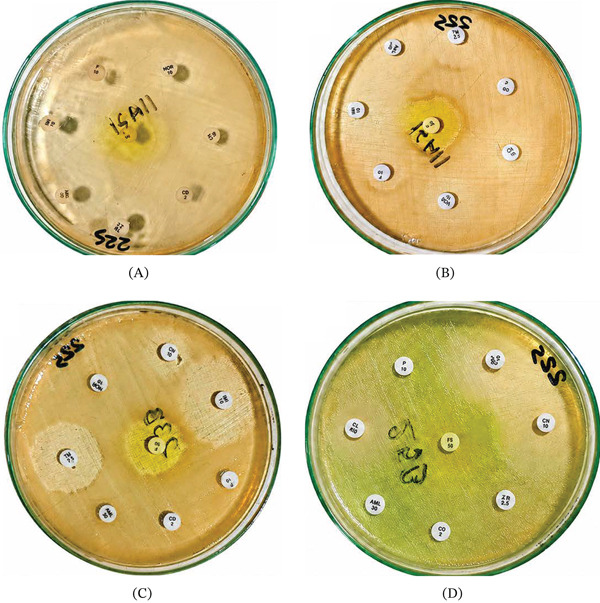
Representative antibiotic susceptibility testing plates showing zones of inhibition. Panels (A–D) show representative disk diffusion antibiotic susceptibility testing plates from clinical isolates obtained from different patients. The plates demonstrate visible zones of inhibition around antibiotic disk, illustrating variation in susceptibility patterns among representative isolates.

AST revealed a high prevalence of MDR among the isolates (Figure 1), with 12 isolates exhibiting resistance to multiple classes of antibiotics. Notably, one *Proteus* spp. isolate demonstrated resistance to all antibiotics included in the tested disk diffusion panel; this was referred to as pan‐resistant within the context of this study.

A high level of resistance was observed against commonly used antibiotics, with 83% and 80% of isolates resistant to ampicillin and clindamycin, respectively. In contrast, most isolates remained susceptible to nitrofurantoin and fosfomycin, with approximately 83% and 70% sensitivity observed, respectively.

**Figure 2:**
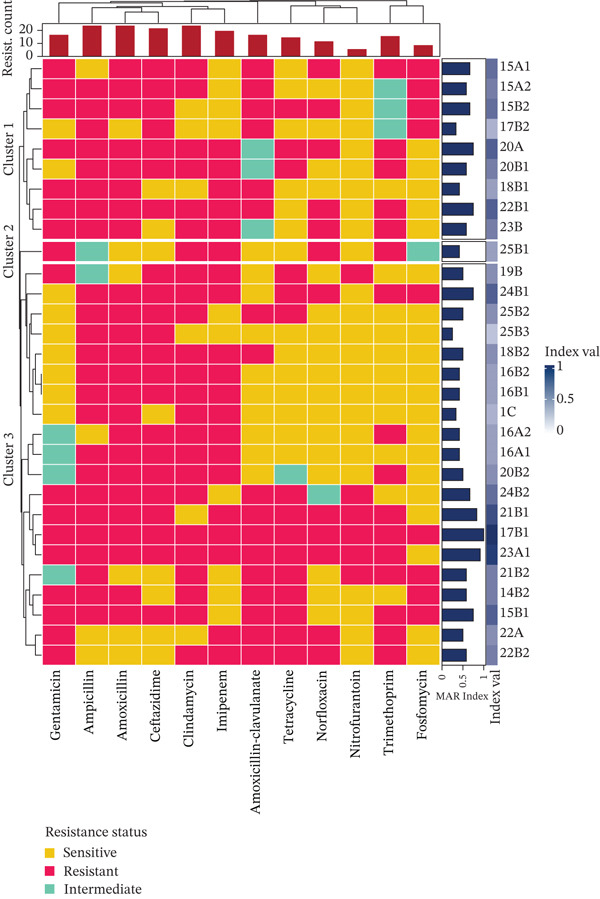
Antimicrobial susceptibility heatmap of uropathogenic isolates. Red indicates resistance, blue indicates sensitivity, and yellow indicates an intermediate response. Rows represent isolates, and columns represent antibiotics. Hierarchical clustering groups isolates with similar resistance patterns. MAR, multidrug antibiotic resistance.

The heatmap (Figure 2) provides a comprehensive visualization of antimicrobial susceptibility patterns across all isolates. Hierarchical clustering revealed distinct grouping of isolates based on their resistance profiles, indicating shared resistance characteristics among certain clusters.

### 3.4. Microbial Abundance

**Figure 3:**
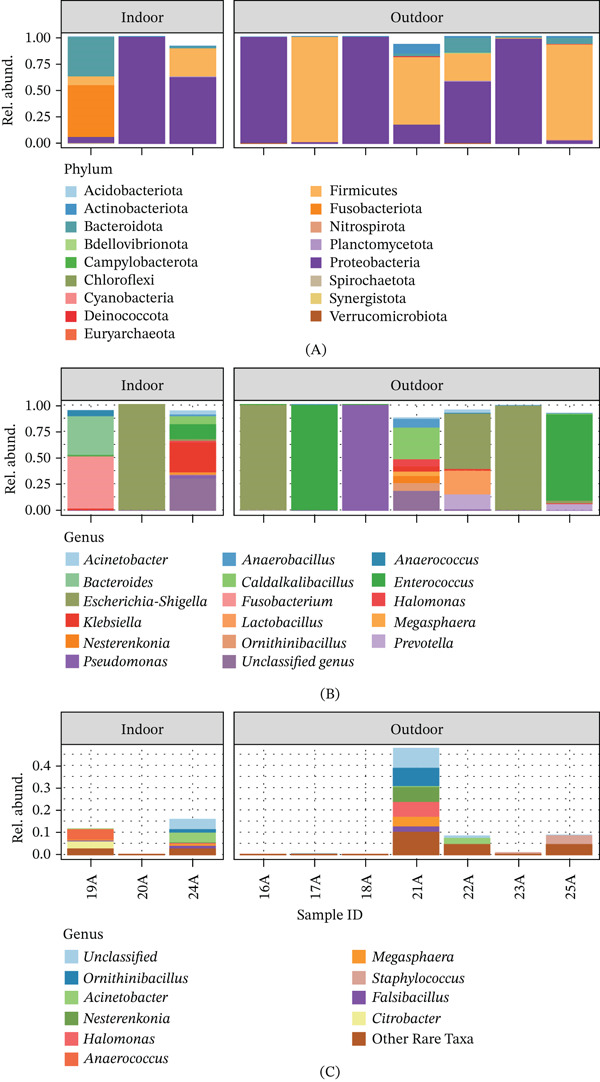
Microbial composition of urine samples. (A) Relative abundance of bacterial communities at the phylum level across inpatient and outpatient samples. Each stacked bar represents an individual sample, with colors indicating different bacterial phyla. (B) Genus‐level relative abundance showing interindividual variation in dominant taxa. Major genera are color‐coded, and each bar corresponds to a single sample. (C) Distribution of low‐abundance genera (≤ 1% relative abundance) across samples. Rare taxa are displayed separately to highlight sample‐specific diversity patterns.

Because microbiome analysis was performed on a relatively small subset of selected patient samples, these findings should be interpreted as exploratory and may not be generalizable to the broader UTI patient population. Phylum‐level microbiome analysis (Figure 3A) demonstrated that the majority of urine samples were dominated by Proteobacteria, indicating a strong presence of typical uropathogens. However, several samples (19A, 21A, 22A, 24A, and 25A) exhibited a more diverse microbial composition, with notable contributions from Fusobacteriota, Actinobacteriota, Firmicutes, and Bacteroidota, suggesting increased community complexity.

At the genus level (Figure 3B), substantial interindividual variation in microbial composition was observed. The genus *Escherichia–Shigella* predominated in samples 16A, 17A, 20A, and 23A, accounting for nearly the entire relative abundance in these samples. *Pseudomonas* was the dominant genus in sample 18A. In contrast, samples 19A, 21A, 22A, and 24A displayed more heterogeneous microbial communities, including genera such as *Bifidobacterium, Lactobacillus*, *Prevotella*, and *Enterococcus*, which are commonly associated with commensal or mixed microbiota.

Low‐abundance genera (≤ 1% relative abundance), presented separately in Figure 3C, constituted a small but detectable fraction of the microbiome. These rare taxa were unevenly distributed across samples, with sample 25A exhibiting the highest diversity of low‐abundance genera.

### 3.5. Microbial Diversity Analysis

**Figure 4:**
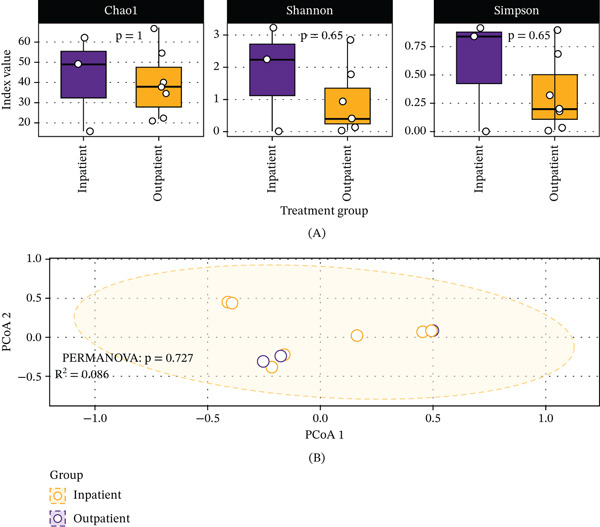
Microbial diversity analysis of urine samples. (A) Alpha diversity indices (Chao1, Shannon, and Simpson) comparing inpatient and outpatient groups. Each point represents an individual sample; boxplots indicate median and interquartile range. No statistically significant differences were observed between groups. (B) Principal coordinates analysis (PCoA) of microbial community composition based on Bray–Curtis dissimilarity. Samples from inpatient and outpatient groups show substantial overlap. PERMANOVA analysis confirms no significant differences between groups (*p* = 0.727, *R* ^2^ = 0.086).

To evaluate microbial richness and evenness between inpatient and outpatient groups, three alpha diversity indices—Chao1, Shannon, and Simpson—were calculated (Figure 4A). Overall, microbial richness was comparable between the two groups, with no statistically significant differences observed (Chao1: *p* = 1.0; Shannon: *p* = 0.65; Simpson: *p* = 0.65). The inpatient group showed a higher median Chao1 index (~49) compared with the outpatient group (~38), indicating a trend toward greater richness; however, this difference was not statistically significant. The outpatient group exhibited a concentration of lower Shannon and Simpson index values, suggesting relatively reduced diversity and evenness, whereas the inpatient samples displayed a wider distribution, indicating greater variability in microbial composition.

PCoA based on Bray–Curtis dissimilarity was performed to assess differences in overall microbial community structure (Figure 4B). The ordination plot demonstrated substantial overlap between inpatient and outpatient samples, with no distinct clustering observed. The first two principal coordinates explained 31% and 19.6% of the total variance, respectively. These findings were supported by PERMANOVA, which showed no significant differences in community composition between the groups (*p* = 0.727, *R* ^2^ = 0.086). Collectively, these results indicate that the urinary microbiome composition does not differ significantly between inpatient and outpatient groups in this cohort.

### 3.6. Functional Pathway Analysis

**Figure 5:**
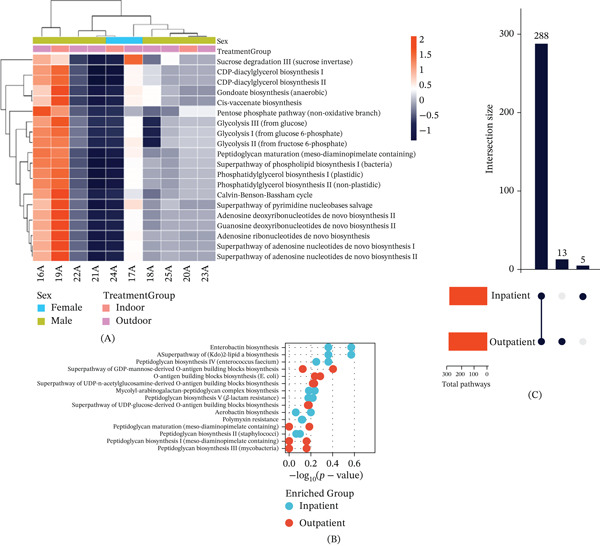
Functional profiling of urinary microbiome. (A) Heatmap of predicted KEGG pathways across samples, showing hierarchical clustering based on functional similarity. No distinct separation between inpatient and outpatient groups is observed. (B) Differential pathway enrichment analysis between inpatient and outpatient groups based on −log10(*p* value). No pathways reached statistical significance (*p* > 0.05). (C) Intersection plot illustrating the number of shared (*n* = 288) and unique pathways between groups.

Functional profiling using PICRUSt2 was performed to predict the metabolic potential of microbial communities based on KEGG orthology annotations (Figure 5). These results represent inferred functional potential, not experimentally confirmed gene expression or metabolic activity. Therefore, pathway differences were interpreted cautiously, and validation by shotgun metagenomics, metatranscriptomics, or targeted assays is needed.

A total of 288 pathways were shared between inpatient and outpatient groups, with 13 pathways uniquely predicted in outpatients and eight pathways in inpatients (Figure 5C). Metabolism‐related pathways, including those involved in carbohydrate metabolism, amino acid metabolism, and energy production, were the most abundant across all samples, as illustrated by the hierarchical clustering heatmap (Figure 5A). The clustering pattern demonstrated no clear separation between inpatient and outpatient samples. Differential pathway analysis (Figure 5B) identified several pathways with relatively higher effect sizes, including carbon metabolism, ABC transporters, and glycolysis/gluconeogenesis; however, none of these differences reached statistical significance (*p* > 0.05).

Notably, inpatient samples showed a relative enrichment of pathways associated with peptidoglycan maturation and O‐antigen biosynthesis, particularly those linked to *Staphylococcus* and *Enterococcus* species. These pathways are related to cell wall structure and virulence‐associated components. In contrast, outpatient samples exhibited a trend toward increased abundance of pathways involved in iron acquisition and environmental adaptation, including enterobactin and aerobactin siderophore biosynthesis. These pathways are typically associated with microbial survival under nutrient‐limited conditions.

## 4. Discussion

This study demonstrates that conventional biochemical methods substantially underestimate the diversity of uropathogens in Bangladesh, whereas molecular approaches reveal a far more complex and clinically relevant urinary microbiome. By integrating enhanced culture techniques with 16S rRNA sequencing, we detected a substantially higher number of bacterial taxa compared with routine diagnostics, highlighting the limitations of standard clinical workflows. Importantly, the dominance of *Escherichia–Shigella* in several samples, coupled with the presence of diverse polymicrobial communities in others, highlights the coexistence of pathogen‐driven and microbiome‐associated dysbiosis. Despite taxonomic variability, the predicted functional potential remained largely conserved across samples, indicating functional redundancy within the urinary microbiome. Together, these findings underscore a critical gap in routine diagnostics and emphasize the need for genomics‐based approaches to improve UTI detection and management.

Several important secondary observations emerge from this study. First, the high prevalence of resistance to commonly used antibiotics such as ampicillin and amoxicillin‐clavulanate aligns with the growing burden of AMR in Bangladesh and similar LMIC settings [3, 4]. The observed MDR patterns further emphasize the need for updated empirical treatment guidelines based on local resistance data. In contrast, the retained effectiveness of nitrofurantoin and fosfomycin supports their continued use as first‐line therapies for uncomplicated UTIs. The retained susceptibility to nitrofurantoin and fosfomycin is clinically relevant because these agents remain important oral treatment options for uncomplicated lower UTIs, particularly when resistance to commonly used antibiotics is high. However, their role in complicated UTIs should be interpreted cautiously, as treatment decisions require consideration of pathogen identity, infection severity, tissue involvement, and patient‐specific clinical factors. Second, the presence of culture‐negative but microbiome‐positive samples suggests that conventional culture methods may fail to detect fastidious or low‐abundance organisms, which may contribute to persistent or recurrent infections and diagnostic uncertainty. Third, the lack of significant differences in microbial diversity between inpatient and outpatient groups indicates that healthcare exposure alone may not fully explain microbiome variation, and that host‐related factors or prior antibiotic exposure may play a more substantial role. Finally, the enrichment of siderophore biosynthesis pathways in outpatient samples and cell wall‐associated pathways in inpatient samples suggests subtle ecological adaptations related to nutrient availability and host interaction, although these trends were not statistically significant and should be interpreted cautiously.

Our findings are consistent with previous studies demonstrating that standard urine culture underestimates microbial diversity and frequently overlooks polymicrobial infections [14, 15]. The predominance of *E. coli* and other Enterobacteriaceae observed in this study aligns with global reports identifying these organisms as leading causes of UTIs [6, 7]. Recent studies from Bangladesh and neighboring South Asian countries provide important regional context for the present findings. A community‐acquired UTI study from Dhaka, Bangladesh, reported *E. coli* as the predominant uropathogen and documented a high burden of MDR among urinary isolates [5]. Similarly, a recent surveillance study from Bangladesh showed increasing resistance among uropathogens to commonly used agents, including ceftazidime, cefuroxime, trimethoprim‐sulfamethoxazole, and amoxicillin‐clavulanate [31]. Another multicenter community‐based study from six districts of Bangladesh reported a higher prevalence of UTI among females and identified *E. coli* as the most common isolate, with substantial resistance to ampicillin and third‐generation cephalosporins [32]. These regional findings are consistent with the high resistance to ampicillin and amoxicillin‐clavulanate observed in our study. Evidence from neighboring countries also supports this pattern: a recent Indian study identified fosfomycin and nitrofurantoin as preferred oral empirical options for uncomplicated *E. coli* cystitis, although regional variation in resistance was noted [33], whereas a study from Pakistan reported high MDR among outpatient uropathogens and emphasized the need for routine culture‐based surveillance before empirical therapy [34]. Additionally, the detection of diverse genera such as *Lactobacillus*, *Prevotella*, and *Bifidobacterium* supports emerging evidence that the urinary tract hosts a complex microbiome rather than being sterile [14]. The observed functional redundancy despite taxonomic variability is also consistent with microbiome studies in other body sites, where distinct microbial communities can maintain similar metabolic functions [35]. Furthermore, the high burden of MDR organisms observed here reflects global trends in increasing resistance among uropathogens, particularly in resource‐limited settings [24].

This study has several limitations that should be considered when interpreting the findings. Because microbiome analysis was performed on a relatively small subset of selected patient samples, these findings should be interpreted as exploratory and may not be generalizable to the broader UTI patient population. First, because this was an exploratory single‐center study with a limited number of samples collected from Dhaka Medical College Hospital, the findings should not be interpreted as nationally representative of uropathogen diversity or AMR patterns in Bangladesh. Instead, the results highlight potential diagnostic gaps and microbial diversity that warrant further investigation. Larger multicenter studies including hospitals from different geographic regions and healthcare settings across Bangladesh are needed to validate these findings and generate nationally representative estimates. Second, the study cohort included a limited number of female patient samples, despite the known higher prevalence of UTIs among females. This sex imbalance may influence the observed microbial diversity and AMR profiles and limits interpretation of the findings in relation to the broader UTI population. Future studies should include larger and sex‐balanced cohorts to better evaluate sex‐specific patterns in uropathogen diversity, urinary microbiome composition, and AMR. Third, the use of 16S rRNA sequencing provides genus‐level resolution in most cases and does not allow precise species‐ or strain‐level identification, which is important for clinical interpretation. Additionally, 16S rRNA gene sequencing provides taxonomic profiling but does not directly identify AMR genes, resistance‐conferring mutations, or strain‐level differences between resistant and susceptible organisms. Therefore, AMR interpretation in this study was based on phenotypic susceptibility testing of cultured isolates. Future studies using whole‐genome sequencing or shotgun metagenomic sequencing will be necessary to link microbial community composition with resistome profiles and specific genetic mechanisms of resistance. Fourth, functional predictions were inferred using PICRUSt2 rather than directly measured through metagenomic or transcriptomic approaches, which may introduce bias in pathway estimation. Fifth, the cross‐sectional design of the study does not allow assessment of temporal dynamics or causality between microbial composition and infection status. Finally, potential confounding factors such as prior antibiotic use, comorbidities, and patient history were not fully captured, which may influence microbial composition and resistance patterns.

This study provides important indication that integrating genomic approaches into routine clinical diagnostics can improve the detection and characterization of uropathogens in Bangladesh. However, implementation in resource‐limited settings will require cost‐effective workflows and capacity building in molecular diagnostics. By revealing hidden microbial diversity and widespread AMR, these findings may improve diagnostic accuracy and guide targeted antibiotic therapy. In resource‐limited settings, where misdiagnosis and inappropriate antibiotic use are common, such approaches could enhance patient outcomes and reduce the burden of resistant infections. Future studies should focus on larger cohorts, longitudinal sampling, and whole‐genome or shotgun metagenomic sequencing to validate these findings and explore pathogen dynamics in greater detail. Overall, transitioning toward genomics‐informed clinical microbiology represents a critical step toward precision diagnostics and improved infectious disease management in Bangladesh and similar high‐burden settings.

## Author Contributions

M.Y. and M.J. developed the protocol and methodology, conceived and coordinated the study, and reviewed the manuscript. M.A.H., T.R., and R. interpreted laboratory data, cleaned and finalized the dataset, performed the descriptive analyses, and prepared the first draft of the manuscript. M.A.H. and T.R. analyzed all the genomic data. M.A.H. was involved in the laboratory work, and analysis of laboratory data and provided intellectual input to the manuscript. R.B.K. collected the clinical samples from the hospital and obtained relevant patient information. M.R. and C.R.A. critically reviewed the manuscript and provided intellectual input.

## Funding

This study was supported by the Department of Microbiology, University of Dhaka.

## Disclosure

The corresponding author affirms that this manuscript is an honest, accurate, and transparent account of the study being reported; that no important aspects of the study have been omitted; and that any discrepancies from the study as planned (and, if relevant, registered) have been explained. All authors reviewed subsequent drafts of the manuscript and approved the final version. All authors had full access to all the data in the study and accepted the responsibility for the integrity of the data, accuracy of the data analysis, and for publication.

## Ethics Statement

This study received ethical clearance from the Committee for Ethical Clearance of Research Proposals, University of Dhaka, under Reference No. 391/Biol.Scs./2025‐2026.

## Conflicts of Interest

The authors declare no conflicts of interest.

## Data Availability

The data that support the findings of this study are available from the corresponding author upon reasonable request.
